# Acetylcholinesterase Inhibitory Activity of Pigment Echinochrome A from Sea Urchin *Scaphechinus mirabilis*

**DOI:** 10.3390/md12063560

**Published:** 2014-06-10

**Authors:** Sung Ryul Lee, Julius Ryan D. Pronto, Bolor-Erdene Sarankhuu, Kyung Soo Ko, Byoung Doo Rhee, Nari Kim, Natalia P. Mishchenko, Sergey A. Fedoreyev, Valentin A. Stonik, Jin Han

**Affiliations:** 1 National Research Laboratory for Mitochondrial Signaling, Department of Physiology, College of Medicine, Cardiovascular and Metabolic Disease Center, Inje University, Busan 614-735, Korea; E-Mails: lsr1113@inje.ac.kr (S.R.L.); juliusryanpronto@gmail.com (J.R.D.P.); s_boloroo_22@yahoo.com (B.-E.S.); kskomd@paik.ac.kr (K.S.K.); bdrhee@hanmail.net (B.D.R.); narikim43@gmail.com (N.K.); 2 Department of Health Sciences and Technology, Graduate School of Inje University, Busan 613-735, Korea; 3 G.B. Elyakov Pacific Institute of Bioorganic Chemistry, Far-Eastern Branch of the Russian Academy of Science, Prospect 100 let Vladivostoku, 159, Vladivostok 690022, Russia; E-Mails: mischenkonp@mail.ru (N.P.M.); fedoreev-s@mail.ru (S.A.F.); stonik@piboc.dvo.ru (V.A.S.)

**Keywords:** acetylcholinesterase, echinochrome A, nitric oxide, sea urchin, uncompetitive

## Abstract

Echinochrome A (EchA) is a dark-red pigment of the polyhydroxynaphthoquinone class isolated from sea urchin *Scaphechinus mirabilis*. Acetylcholinesterase (AChE) inhibitors are used in the treatment of various neuromuscular disorders, and are considered as strong therapeutic agents for the treatment of Alzheimer’s disease (AD). Although EchA is clinically used to treat ophthalmic diseases and limit infarct formation during ischemia/reperfusion injury, anti-AChE effect of EchA is still unknown. In this study, we investigated the anti-AChE effect of EchA *in vitro*. EchA and its exhausted form which lost anti-oxidant capacity did not show any significant cytotoxicy on the H9c2 and A7r5 cells. EchA inhibited AChE with an irreversible and uncompetitive mode. In addition, EchA showed reactive oxygen species scavenging activity, particularly with nitric oxide. These findings indicate new therapeutic potential for EchA in treating reduced acetylcholine-related diseases including AD and provide an insight into developing new AChE inhibitors.

## 1. Introduction

Acetylcholine (ACh) is a neurotransmitter in the central nervous system [1] that can induce vasorelaxation, possibly through the modulation of ion channels affecting membrane potential in smooth muscle cells [2]. Besides its role as a neurotransmitter, ACh confers protection against myocardial stresses such as ischemia/reperfusion [3,4]. Acetylcholinesterase (AChE; E. C. 3.1.1.7) is a serine hydrolase belonging to the carboxylesterase family of enzymes, which hydrolyzes acetylcholine (ACh) into choline and acetic acid. AChE inhibitors or anti-cholinesterases augment both level and duration of neurotransmitter activity of ACh at the cholinergic synapse, as well as in vascular and other target tissues [1,5]. The etiology of Alzheimer’s disease (AD) is also linked to a possible role of β-amyloid deposition, oxidative stress, and inflammation [6]; hence, development of drugs that can also target these pathological traits would be vital in formulating AD treatment strategies. Nitric oxide (NO) plays important role in central nervous system and blood vessel but pathological level of NO can lead to promotion of development of AD through nitrosative stress [7]. The pathogenesis of AD is associated with loss of cholinergic neurons and, consequently, a reduced availability of ACh; thus, anti-AChE drugs are available drug for mitigating some AD-related symptoms and thus improve cognition through enhanced activation of synapses [1,8]. The application of anti-AChE drugs is not limited to the treatment of AD, since anti-AChE drugs are applied to treat various forms of dementia including Parkinson’s Disease and dementia [9]. However, there are only a few clinically approved AChE inhibitors (donepezil, rivastigmine, and galantamine), whose long-term effects are still debatable [10].

Numerous inhibitors against AChE are either isolated from natural sources [8,11] or are chemically synthesized [1]. Plants, marine animals and terrestrial microbes have been used sources of new compounds with anti-AChE activity. Identified compounds can be categorized as (1) alkaloids; (2) coumarins; (3) flavonoids; (4) quinones; (5) stilbenes; (6) terpenic compounds, and xanthones [8]. It has been known that the quinonoid group plays an important role in AChE inhibition, since AChE can be inactivated by dopamine autoxidation [12]. However, there are few studies concerning the inhibition of AChE by quinone compounds (benzoquinone and naphthoquinones) [12,13].

Echinochrome A (EchA), occurring in the sea urchin *Scaphechinus mirabilis* (Agassiz) and other species of sea urchins [14], is a dark-red pigment and have a naphthazarin fragment, which makes it suitable for metal ion chelation [15]; and also possesses three hydroxyl groups for free-radical scavenging via a homolytic reaction [14,16]. As polyhydroxynaphthoquinone derivatives, EchA has cardioprotective capacity, limiting infarct formation during ischemia/reperfusion injury [17,18,19]. It also possesses an inhibitory effect on dopamine-beta-hydroxylase [20]. Histochrome^®^ is a recently developed water-soluble derivative of its lipophilic parent, EchA (6-ethyl-2,3,5,7,8-pentahydroxy-1,4-naphthoquinone) [14]. In clinical application, this drug has also been widely observed in ophthalmology for eliminating inflammation in the retina, vascular membrane and cornea of the eye [21,22]. However, previous studies on these effects of EchA suggest that its activity is not entirely attributable to its direct anti-oxidant effects. AChE inhibitors, aside from their anti-oxidant properties, have also been reported to induce an anti-inflammatory response by directly inhibiting cytokine release, as observed in microglia and monocytes [6]. Therefore, the anti-inflammatory properties of EchA should also be examined.

To date, there are no known reports regarding the inhibitory activity of EchA on AChE. This prompted us to determine the inhibitory effect of EchA on AChE and its possible mode of action. ACh-mediated vasorelaxation in smooth muscle cells may be related to the production of nitric oxide (NO) in endothelial cells [2], which, when unregulated, can lead to abrupt vascular complications and severe inflammatory response [23]. In this regard, the scavenging potential of EchA was determined by NO production of an exogenous NO donor, sodium nitroprusside (SNP) [24]. In this report, the AChE inhibition and NO scavenging effect of EchA *in vitro* are presented.

## 2. Results and Discussion

### 2.1. Cytotoxicity of Echinochrome A

**Figure 1:**
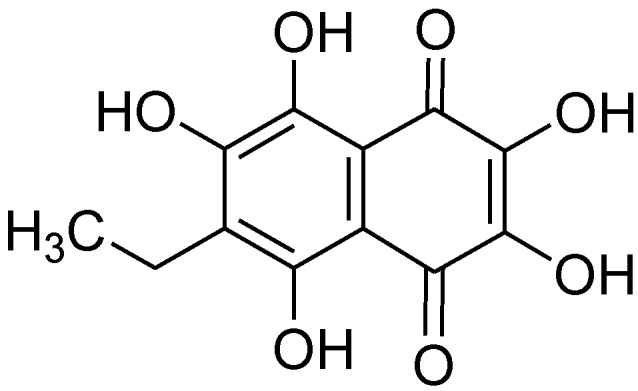
The chemical structure of echinochrome A (EchA) (6-ethyl-2,3,5,7,8-pentahydroxy-1,4-naphthoquinone, FW = 266.2) comes from a class of naturally occurring polyhydroxynaphthoquinones. This compound has a red-brown color.

**Figure 2:**
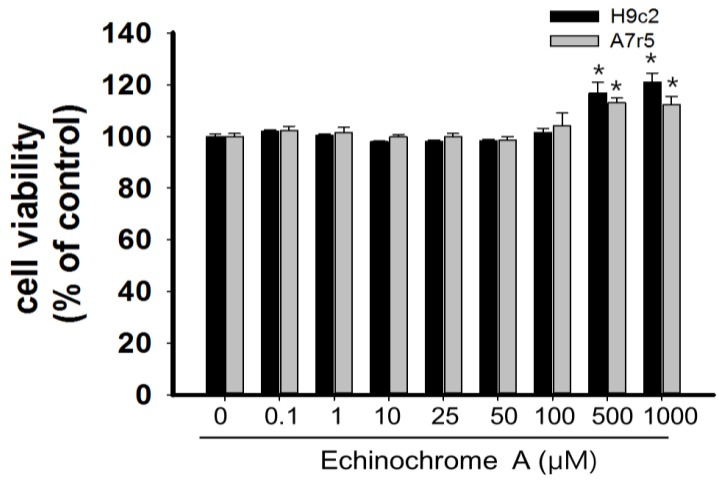
Determination of cell cytotoxicity of echinochrome A on H9c2 and A7r5 cells. Cells were treated with echinochrome A for 24 h and cell viability was determined by MTT assay. There was no significant cytotoxicity on both cell lines. Cell viability was calculated as % of untreated control. Values are expressed as mean ± SEM (*n* = 10). * *p* < 0.05 *vs.* untreated control.

EchA was obtained from Pacific Institute of Bioorganic Chemistry, Far East Branch of the Russian Academy of Sciences (Figure 1). EchA did not show significant toxicity on A7r5 cells (rat aortic vascular smooth muscle cell line) [25] and H9c2 cells (rat cardiomyoblasts) [26] even up to 100 μM for 24 h (Figure 2). However, EchA above 500 μM slightly increased cell viability. The exact reason for this is unclear but this increase in cell viability may be related to the cellular protective role of EchA in H9c2 and A7r5 cells.

**Figure 3:**
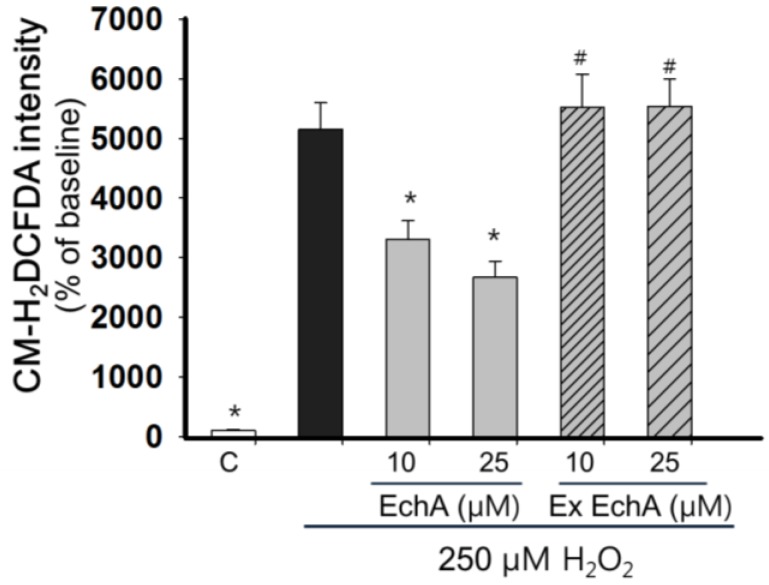
Determination of ROS-scavenging activity of EchA and exhausted EchA. H9c2 cells were seeded on a 48 well culture plate. After confluence, cells were washed twice with Tyrode solution and then stained with 10 μM CM-H_2_DCFDA for 30 min. After washing cells with Tyrode, cells were exposed to 250 μM H_2_O_2_ in the presence or absence of EchA (10 and 25 μM). The changes in CM-H_2_DCFDA intensity were measured with a fluorescence plate reader (SpectraMax M2e, Molecular Devices, Sunnyvale, CA, USA) at before and 10 min after adding the H_2_O_2_. Data were calculated as a % increase of value at baseline (*n* = 8). EchA: echinochrome A, ex EchA: exhausted EchA, * *p* < 0.05 *vs.* 250 μM H_2_O_2_, # *p* < 0.05 *vs.* the same concentration of EchA.

EchA is regarded as an antioxidant [27] and may quickly lose its antioxidant potential upon exposure under room air and/or light. Exhausted form of EchA was prepared by exposing EchA to room air under light for 48 h. To test diminished oxidative activity of exhausted EchA, H_2_O_2_-scavenging activity of EchA and exhausted EchA were compared after staining with CM-H_2_DCFDA, a fluorescence probe for reactive oxygen species (ROS), on H9c2 cells. As shown in Figure 3, 250 μM H_2_O_2_ significantly increased the CM-H_2_DCFDA fluorescence and EchA (10 and 25 μM) showed significant ROS scavenging activity. However, exhausted EchA did not show any ROS-scavenging activity.

**Figure 4:**
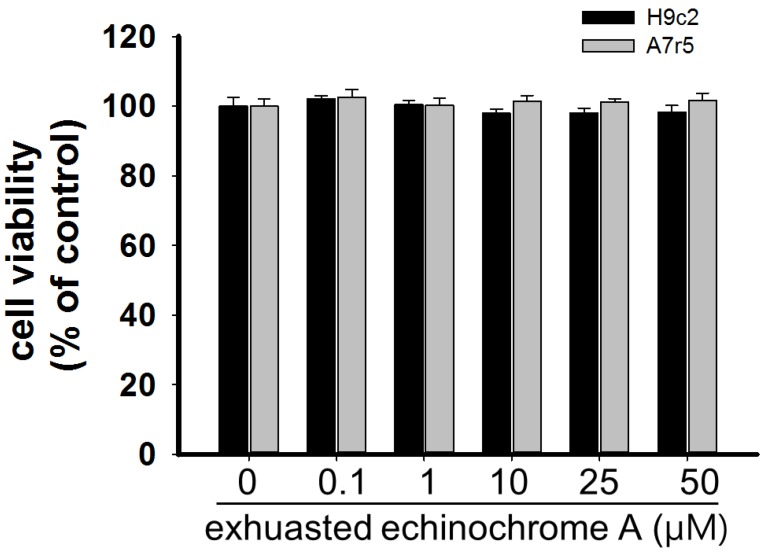
Determination of cell cytotoxicity of exhausted echinochrome A on H9c2 and A7r5 cells. Exhausted form of EchA was prepared by exposure of EchA to room air under light for at least 48 h. The exhausted form of EchA lost its H_2_O_2_-scanvenging capacity (Figure 3) but did not show any cytotoxic effect on H9c2 and A7r5 cells up to 50 μM (*n* = 10). Cell viability was determined by MTT assay and calculated as % of untreated control. There were no statistical significances (*p* > 0.05).

To exclude the possible transformation of EchA into toxic compounds under room air, cytotoxicity test was performed with an exhausted form of EchA. As shown in Figure 4, there was no difference in cellular toxicity on either of the two cell lines in the presence of exhausted EchA up to 50 μM. This result implies that EchA is less toxic to cells even when lost its antioxidant capacity.

### 2.2. Inhibitory Effect of Echinochrome A on Acetylcholinesterase

**Figure 5:**
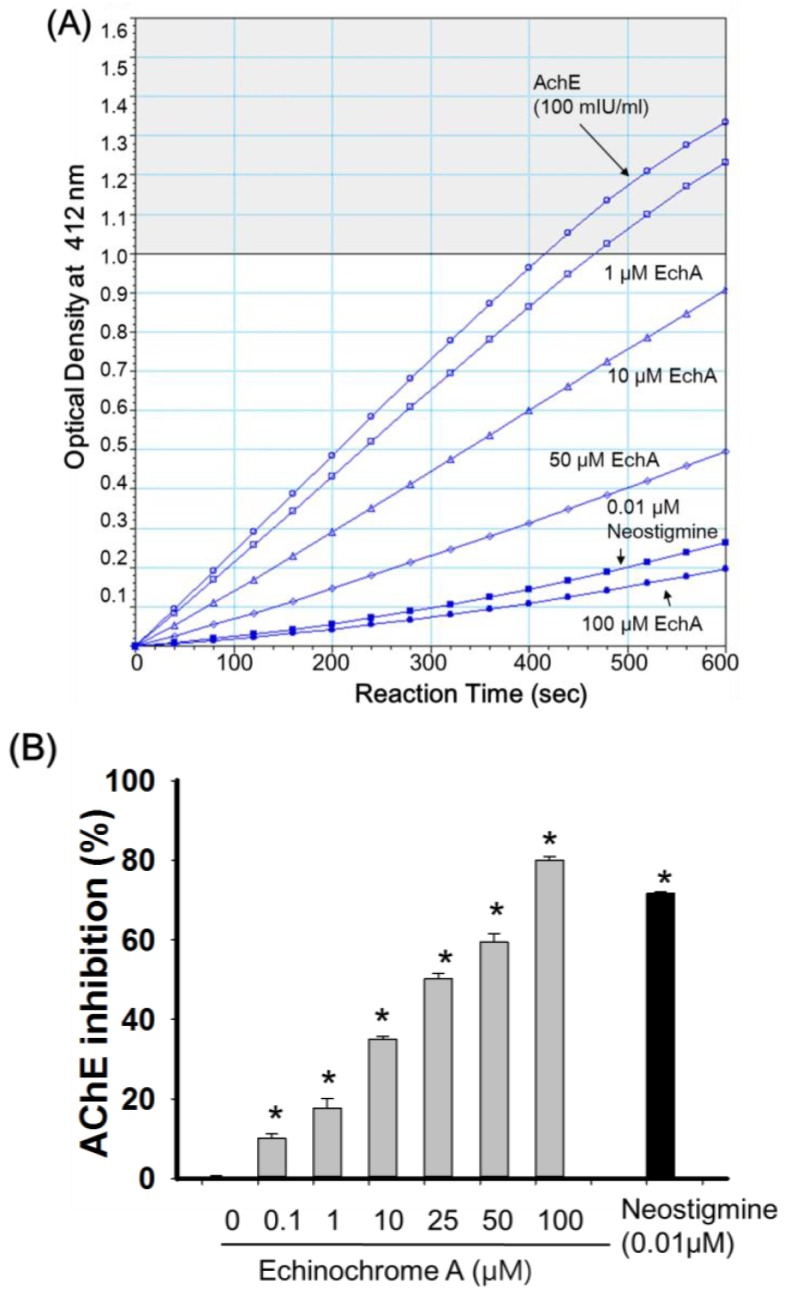
Anti-acetylcholinesterase activity of echinochrome A. (**A**) Representative product formation image as a function of time at different concentration of EchA (*n* = 9). Neostigomine was used as positive control of anti-AChE activity. The activity of AChE (100 mIU/mL) alone was used as a control for determining the anti-AChE effect of EchA; (**B**) Dose-dependent anti-AChE activity of EchA. The AChE inhibitory effect of EchA was calculated as % of AChE alone from Figure 5A. Values are expressed as mean ± SEM. AChE: acetylcholinesterase, EchA: echinochrome A, * *p* < 0.05 *vs.* AChE alone.

Next, we tested the anti-acetylcholinesterase activity of EchA. To obtain reliable AChE activity, purified AChE (100 mU/mL) from a commercially available AChE activity assay kit (AAT Bioquest, Sunnyvale, CA, USA) was used. The activity of AChE was determined by thiocholine formation of DTNB (5,5′-dithiobis-2-nitrobenzoic acid) from the hydrolysis of ACh [28]. Neostigomine, that is used as a parasympathomimetic drug but has severe side effects such as headache, brow pain, blurred vision [29] was used as a positive control. As shown in Figure 5A, EchA showed anti-AChE activity in a dose-dependent manner. Based on Figure 5B, the half maximal AChE inhibition of EchA was calculated through linear regression analysis and assumed to be 16.4 μM. Among quinone-related compounds, it has been reported that the IC_50_ values of sargaquinoic acid and mansonones E are 23.3 and 23.5 μM, respectively [12]. In our experimental setting, the anti-AChE activity of EchA was better than other quinone-related compounds, sargaquinoic acid and mansonones E [30]. However, direct comparison of compounds on anti-AChE activity needs caution, since the inhibitory concentrations of tested compounds are dependent on incubation time, reaction conditions, source of enzymes and assaying method [8].

### 2.3. Mode of Inhibition of Echinochrome A on Acetylcholinesterase

**Figure 6:**
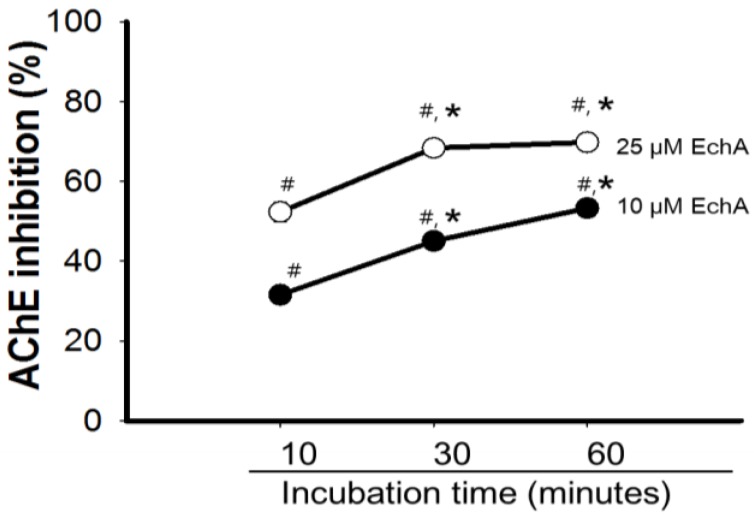
Anti-acetylcholinesterase activity of echinochrome A with incubation time. Inhibitory activity of EchA was assessed based on incubation time of AChE with EchA before adding the ACh. The activity of AChE alone at different incubation time (control) was set at 100%. The inhibitory effects of EchA on AChE were expressed as % of respective control. Data are mean ± SEM (*n* = 5). AChE; acetylcholinesterase, EchA; echinochrome A, # *p* < 0.05 *vs.* AChE alone at different incubation time, * *p* < 0.05 *vs.* AChE with EchA at 10 min incubation.

**Figure 7:**
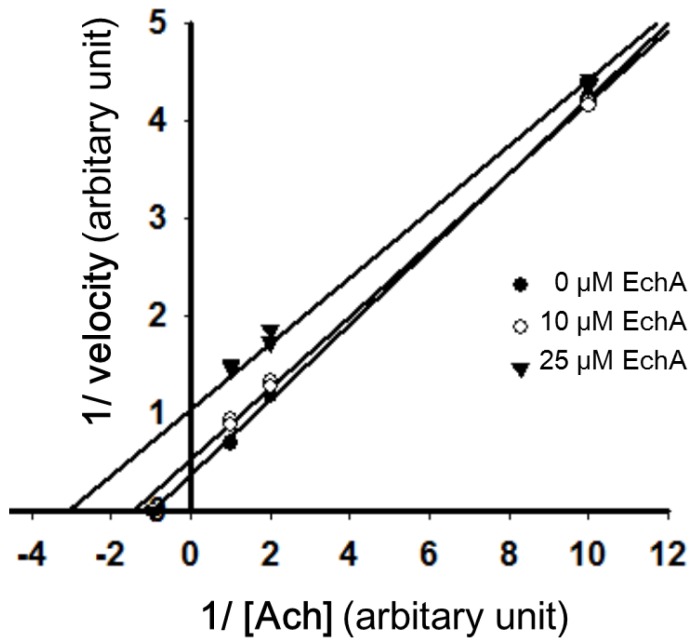
Lineweaver-Burk plot of 1/v *vs.* 1/[ACh] in the presence or absence of EchA. Slope indicates *K_m_*/V. The *V_max_* (*y*-intercept) and *K_m_* (*x*-intercept) value of AChE as plotted against [ACh] were decreased significantly in the presnec of EchA. EchA; echinochrome A.

**Figure 8:**
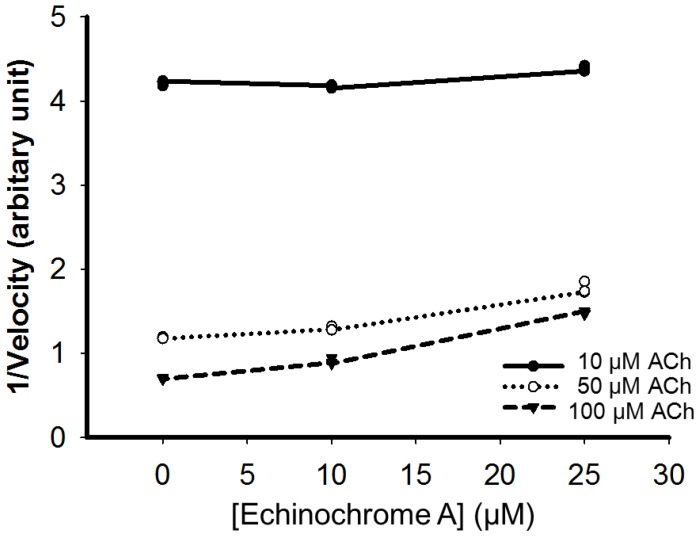
Dixon plot. The *V_max_* value of AChE was plotted against different doses of EchA at multiple fixed Ach concentrations. The *K_i_* value (*x*-intercept) could not be obtained because theslopes are parallel. ACh: acetylcholine.

Inhibition of AChE can be achieved in a competitive, non-competitive/mixed, and uncompetitive manner [31,32]. Uncompetitive AChE inhibitors inhibit AChE not through competition for the binding site of the naturally occurring substrate but through binding to the enzyme-substrate complex [1,33]. The inhibitory effect of a uniform dose of EchA revealed to be dependent on incubation time with AchE, suggesting that EchA may serve as a weak irreversible AChE inhibitor (Figure 6). To determine whether AChE inhibition by EchA is competitive or non-competitive, the inhibitory action of EchA was measured against different concentrations of Ach (Figure 7). The *K_m_* and *V_max_* values were calculated through the Lineweaver-Burk plot. As shown in Figure 7, the *V_max_* values of 0, 10, and 25 μM EchA were 2.76, 1.81, and 0.88, respectively. The *K_m_* values of 0, 10, and 25 μM EchA were 1.07, 0.65 and 0.21, respectively. The decreases in both *K_m_* and *V_max_* suggest that the EchA serves as an uncompetitive inhibitor on AChE. Next, Dixon plots was applied for measuring the *K_i_* value [34]. As shown in Figure 8, there were no changes in slope and thus *K_i_* value could not be obtained. Based on Figure 7 and Figure 8, these results suggest that the EchA on AChE works as an uncompetitive inhibitor. This uncompetitive mode of anti-AChE activity of EchA may provide an insight in developing new anti-AChE drugs.

### 2.4. Nitric Oxide Scavenging Effect of Echinochrome A

**Figure 9:**
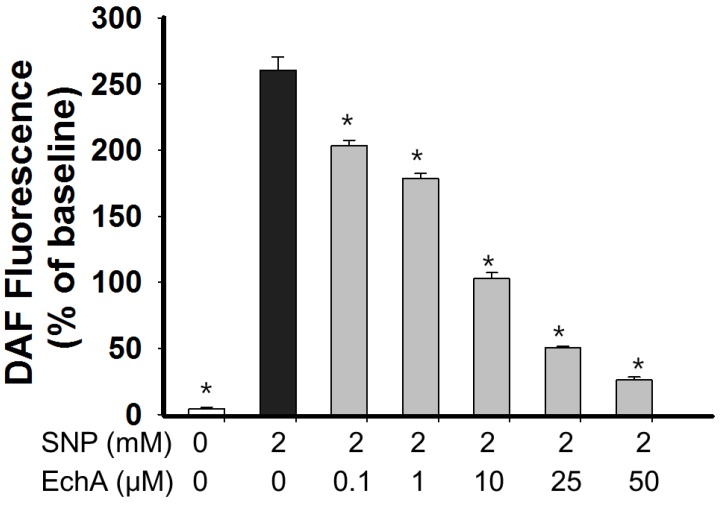
NO scavenging activity of echinochrome A. A7r5 cells were stained with DAF-FM (10 μM) for 15 min in Tyrode buffer and cells were exposed to different concentrations of EchA after washing. SNP was used as a NO donor. After treatment with 2 mM SNP for 30 min, NO production, indicated by an increase in DAF-FM fluorescence, was determined using a fluorescence microplate reader. Values from without exposure to 2 mM SNP are set at 100% and then expressed as % of baseline (mean ± SEM; *n* = 9) after 30 min incubation. EchA; echinochrome A, SNP; sodium nitroprusside, * *p* < 0.05 *vs.* 2 mM SNP.

Nitric oxide (NO) plays a crucial role in the maintenance of vascular tone and is involved in various signaling pathways, but excessive NO levels can also lead to inflammation [35]. It has been suggested that there is a close link between the cholinergic system and inflammation and thus anti-AChEs with anti-inflammatory potential will be more favorable in treating AChE-depletion related pathological condition including AD [6]. From this point of view, EchA will serve as a good therapeutic candidate, since EchA has an anti-AChE activity and anti-oxidant potential which is helpful in suppressing inflammatory mediators such as ROS and NO. To test the NO scavenging effect of EchA, A7r5 cells were stained with a NO-sensitive fluorescence probe DAF-FM (4-amino-5-methylamino-2′,7′-difluorofluorescein; 10 μM) and then NO production was determined in the presence of different doses of EchA (0–50 μM) after exposure to 2 mM sodium nitroprusside (SNP) [24]. After SNP treatment, DAF-FM fluorescence increased with time. There was no significant difference in NO levels when EchA alone (0–50 μM) was exposed to A7r5 cells for 30 min (*n* = 9, *p* > 0.05). Cellular NO on A7r5 cells greatly increased following exposure to SNP (control, 4.0% ± 1.5%; 2 mM SNP, 260.5% ± 10.0%; *p* < 0.05) and NO production continuously increased with time. As shown in Figure 9, EchA showed NO-scavenging activity in a dose-dependent manner. However, EchA did not completely scavenge the NO produced by 2 mM SNP, even at 50 μM. EchA has an iron chelating capacity [15]; thus, there is a possibility that NO production may be suppressed through chelation of the cyanide moiety of SNP in the presence of EchA. However, NO decomposes spontaneously from SNP, and nitrite production from SNP in the presence of iron chelator defroxamine was unchanged [36]. Therefore, the decrease in DAF fluorescence in the presence of EchA may reflect the immediate scavenging of NO by EchA. This excessive NO eliminating capacity of EchA may be applicable in certain pathological conditions, such as NO-mediated inflammatory response. It is speculated that EchA may not produce a direct and immediate vasorelaxation effect since ACh- or other stimulus-mediated NO production could be suppressed in the presence of EchA.

Taken together, EchA exhibits an irreversible mode of anti-AChE activity (IC_50_ ≈ 16.4 μM) and also performs a NO scavenging activity, which may also reflect an anti-inflammatory role for EchA as an AChE inhibitor. These biological capacities of EchA may give insight into the development of new AChE inhibitors that also target other etiological causes of AD, and with less side effects, which may enlarge its application in the clinical setting.

## 3. Experimental Section

### 3.1. Experimental General

We used Histochrome^®^ containing 0.02% or 1% echinochrome A (PN002363/02) produced by Pacific Institute of Bioorganic Chemistry, Far East Branch of the Russian Academy of Sciences. Amplite™ colorimetric acetylcholinesterase assay kit was obtained from AAT Bioquest (Sunnyvale, CA, USA). The 4-amino-5-methylamino-2′,7′-difluorofluorescein diacetate (DAF-FM) was used as specific NO indicator [37] and was purchased from Molecular Probes (Invitrogen, Carlsbad, CA, USA). Neostigmine bromide was purchased from Sigma (St. Louis, MO, USA) and used as a positive inhibitor of AChE. All other chemicals are obtained from Sigma (St. Louis, MO, USA).

### 3.2. Cell Culture

H9c2 (rat myoblast cell line, ATCC CRL-1446) and A7r5 cells (rat aortic vascular smooth muscle cell line, ATCC CRL-1444) were cultured in Dulbecco’s Modified Eagle’s Medium (DMEM) with high glucose supplemented with 10% fetal bovine serum (FBS) and 1% penicillin/streptomycin (100 units/mL penicillin and 100 μg/mL streptomycin) (Gibco, Carlsbad, CA, USA) until confluence.

### 3.3. Cell Cytotoxicity Test

H9c2 and A7r5 cells were seeded at a concentration of 2 × 10^4^ cells/well in 96-well tissue culture plates (Nunc, Rockford, IL, USA) and treated with different concentrations of EchA (0–100 μM) for 24 h. After treatment, cell viability was assessed by incubating the cells with 250 μg/mL of MTT [3-(4,5-dimethylthiazol-2-yl)-2,5-diphenyltetrazolium bromide] for another 1 h [38]. The MTT formazan produced by viable cells was dissolved in dimethylsulfoxide (DMSO) and a microplate reader SpectraMax M2e (Molecular Device, Sunnyvale, CA, USA) was used to measure the optical density (OD) at 560 nm. The total formazan formation is proportional to the number of viable cells in the culture and the OD of untreated cultures was set at 100%. At least three independent experiments were performed.

### 3.4. Measurement of Reactive Oxygen Species Level

H9c2 cells were seeded on a 48-well culture plate and incubated until confluence. After washing cells twice with Tyrode solution containing (in mM) NaCl 135, KCl 5.4, HEPES 5, NaH_2_PO_4_ 0.33, MgCl_2_ 0.5, glucose 16.6, and CaCl_2_ 1.8, pH 7.4, cells were stained with 10 μM CM-H_2_DCFDA (excitation/emission = 490 nm/530 nm) dissolved in Tyrode solution for 30 min in a 37 °C incubator. Cells were resuspended in Tyrode solution and then treated with 0, 10, 25 μM of EchA for 10 min. Baseline fluorescence intensity was measured and then cells were treated with 250 μM H_2_O_2_ for 10 min. The changes in CMH2DCFDA intensity was measured with a fluorescence plate reader (SpectraMax M2e, Molecular Devices, Sunnyvale, CA, USA) at before and 10 min after adding the H_2_O_2_. Data were calculated as a % increase of value with respective baselines (*n* = 8).

### 3.5. Anti-Acetylcholinesterase Activity Assay

The inhibitory activity of EchA on AChE was determined by Amplite™ colorimetric acetylcholinesterase assay kit with slight modification. Purified AChE (50 Units/mL) was dissolved in 0.1% Bovine serum albumin. The enzyme (100 mU/mL), the co-substrate 5,5-dithiobis-2-nitrobenzoic acid (200 μM), and the different concentrations of EchA (0–100 μM) were incubated for 10 min at 37 °C. The mixture was dissolved in phosphate buffer (100 mM Na_2_HPO_4_, 100 mM NaH_2_PO_4_, pH = 7.4) to a final volume of 50 μL per well. The reaction was started with 50 μL of acetylcholine (200 μM). Neostigomine bromide (IC_50_ = 0.006 μM) was used as a positive control for the inhibition of AChE. However, incubation time in AChE containing mixture and concentration of ACh were variable depending on experimental settings. The developing yellow color was measured at 412 nm over 20 min with 50-s interval using a Molecular device microplate reader SpectraMax M2e (Sunnyvale, CA, USA). The resulting AChE activity was obtained as a velocity (*V_max_*). AChE activity without any compounds were set at 100% and expressed as a percentage of AChE alone. The inhibitory mode of EchA on AChE was determined by Lineweaver-Burk and Dixon plot [34].

### 3.6. Determination of Nitric Oxide Production

A7r5 cells were seed at the density of 2 × 10^4^ cells/well in a 48 well tissue culture plates (Nunc, Rockford, IL, USA). After washing twice with Tyrode solution containing (in mM) NaCl 135, KCl 5.4, HEPES 5, NaH_2_PO_4_ 0.33, MgCl_2_ 0.5, glucose 16.6, and CaCl_2_ 1.8, pH 7.4, cells were loaded with 10 μM DAF-FM for 20 min at 37 °C. After washing once with Tyrode solution, the cells were treated with different doses of EchA (0–50 μM) for 10 min and then 2 mM sodium nitroprusside (SNP) was applied in the cells. The intensity of DAF-FM fluorescence (excitation/emission; 495/515 nm) was measured using SpectraMax M2e (Molecular Devices, Sunnyvale, CA, USA) with scan mode at directly after adding SNP (baseline) and 30 min after SNP exposure. Values were obtained as a relative fluorescence unit and expressed as a percentage of the baseline. The SNP-untreated group was used as a negative control.

### 3.7. Statistical Analysis

All values are expressed as the mean ± SEM of at least three independent experiments. Significance was determined using one-way ANOVA followed by Dunnett’s Method or *t*-test (Systat Software Inc., San Jose, CA, USA), and *p* < 0.05 was considered significant.

## 4. Conclusions

The results obtained from this study clearly indicate that EchA has a powerful anti-AChE activity exhibiting an irreversible and uncompetitive mode of inhibition. EchA and its exhausted form have less cytotoxicy on H9c2 and A7r5 cells. Unlike other quinones, EchA has nitric oxide scavenging activity and anti-oxidant potential. However, the elucidation of chemical structure related to anti-AChE with uncompetitive mode of EchA should be further investigated through comparison with other known AChE inhibitors. Taken together, the anti-AChE and anti-oxidant activities of EchA might be applied in treating ACh-limited diseases and the characteristic of EchA may provide an insight into developing a new valuable drug.
